# Sugar-Sweetened Beverage Consumption Status and Its Association with Childhood Obesity among Chinese Children Aged 6–17 Years

**DOI:** 10.3390/nu13072211

**Published:** 2021-06-27

**Authors:** Qian Gan, Peipei Xu, Titi Yang, Wei Cao, Juan Xu, Li Li, Hui Pan, Wenhua Zhao, Qian Zhang

**Affiliations:** National Institute for Nutrition and Health, Chinese Center for Disease Control and Prevention, Beijing 100050, China; gqian212@outlook.com (Q.G.); xupp.122@163.com (P.X.); didi2001@163.com (T.Y.); caow2012@126.com (W.C.); xujuan@ninh.chinacdc.cn (J.X.); lychee317@163.com (L.L.); panhui0204@sina.com (H.P.); zhaowh@chinacdc.cn (W.Z.)

**Keywords:** school age, sugar-sweetened beverage, Chinese children, overweight/obesity

## Abstract

Objective: There is a remarkable growth in sugar-sweetened (SSB) production and obesity prevalence among school-aged children in China. This paper describes SSB consumption and its association with obesity among Chinese children aged 6–17 years in 2012. Methods: in total, 25,553 children aged 6~17 years enrolled in the China Nutrition and Health Surveillance 2010–2013 were included in this study. Data of SSB consumption frequency and quantity were obtained from a food frequency questionnaire, and the children’s nutritional status was assessed. Multivariate logistic regression was used to evaluate the association between SSB consumption and obesity status. Results: SSB intake was estimated as 181.0 g/day, occurring 2.2 times/week. Older children, males, children from urban areas, and children with higher socioeconomic status were more likely to consume SSBs. Children who consumed SSBs 1~<5 times/week (11.7%) and >5 times/week (12.9%) were more likely to be overweight/obesity than those who consumed SSBs less than once/week. Conclusion: SSB consumption was common among Chinese school-aged children, especially among males, older children, and children from urban areas. High consumption of SSBs was associated with a higher prevalence of overweight/obesity. Actions and plans are required to reduce SSB consumption and control childhood obesity in China.

## 1. Introduction

Childhood obesity became an increasingly serious public health problem worldwide. According to a report by the World Health Organization, from 1975 to 2016, the prevalence of overweight or obesity children and adolescents aged 5~19 years increased more than fourfold from 4% to 18% globally [1]. In United States, the prevalence of obesity among children and adolescents aged 2~19 years increased from 10% in 1988–1994 to 17.2% in 2013~2014 [2]. Once common only in high-income countries, obesity is now also prevalent in low- and middle-income countries. In China, according to the Chinese Residents Chronic Diseases and Nutrition Surveillance, the prevalence of overweight/obesity among Chinese children aged 6~17 years was 15% in 2012 [3]. Overweight/obesity prevalence increased by 9.2% during the ten-year period from 2002 to 2012 and increased to 19% in 2016 [3,4].

In recent years, beverage consumption, in particular sugar-sweetened beverages (SSBs), was reported by a number of studies to be highly related to the increasing prevalence of obesity and weight gain [5,6,7,8,9,10,11]. Children and adolescents were reported to consume more SSBs than those in other age groups [12], and the increasing trend of SSB consumption was a major concern during past decades worldwide [13,14,15]. In the United States, the average caloric intake from SSBs among children aged 2~19 years increased from 204 in 1994 to 224 kcal/day in 2004, and then declined to 132.5 in 2014 [14,15]. In Korea, children and adolescents’ beverage intake steadily increased from 1998 to 2016~2018 [16]. A study from China covering 4 cities indicated that SSB consumption among 8–14-year-old children in urban areas increased from 329 to 715 mL/d from 1998 to 2008 [17]. Meanwhile, the annual production of beverages in China increased from 9.6 million tons to 64.2 million tons from 1998 to 2008, and then increased to 177.6 million tons in 2019 [18]. However, nationwide studies of SSB consumption among Chinese school-aged children over the last two decades are limited. This study aimed to describe SSB consumption among school-aged children in China in a nationwide survey and discuss its association with obesity status.

## 2. Materials and Methods

### 2.1. China Nutrition and Health Surveillance

The China Nutrition and Health Surveillance (CHNHS) was a cross-sectional survey conducted by the National Institute for Nutrition and Health (NINH) from 2010 to 2013. A probability proportionate to size sampling design was used for the selection of participants in the CHNHS. The survey covered 31 provinces in China. In total, 150 monitoring sites were selected and divided into 4 strata (large cities, small to medium cities, general rural areas, and poor rural areas) based on their economic characteristics and social development. Six communities were selected from each monitoring site, and 75 households were selected from each community. Twenty children from each year of age in each household were chosen, and in the case of insufficiency, children were supplemented with those from nearby schools. In total, 183,137 participants aged 6~17 years were enrolled in the CHNHS.

Ethics approval was obtained from the ethics committee of the China Centre for Disease Control. All participants provided informed consent.

### 2.2. Analytic Sample

Data from this study were extracted from the CHNHS, and only children aged 6~17 years were included. Household basic information questionnaire, food frequency questionnaire (FFQ), and physical examination were used in this study.

SSB consumption frequency and quantity, energy intake, fat intake and carbohydrate intake were obtained from the FFQ in the CHNHS. Children from each household were asked in a personal interview to recall their food intake frequency and quantity during the past 12 months. All participants were asked at first whether they consumed beverages during the past 12 months from the day of the survey, and then to recall the frequency of consumption and quantity consumed. Seven kinds of beverages on Chinese market were included: carbonated beverage, 100% fresh juice, juice, lactobacillus beverage, formulated milk beverage, coffee, and tea. In this study, we excluded 100% fresh juice and divided the other beverage types into four groups: carbonated beverages, juice beverages, milk beverages (including lactobacillus and formulated milk beverages), and coffee and tea beverages. Dairy (including milk, flavored milk, yogurt, etc.) were not included in this study. SSB consumption frequency was divided into three categories: “<once/week,” “1 to <5 times/week,” and “≥5 times/week.” SSB consumption quantity was also divided into three categories: “0 to <150 g/day,” “150 to <500 g/day,” and “≥500 g/day.” Energy intake, fat intake, and carbohydrate intake were calculated based on food consumption in FFQ. Energy intake was then divided into three categories: “<1500 kcal/day”, “1500 to <2500 kcal/day”, and “≥2500 kcal/day”.

Information about date of birth, sex, region, household annual income, physical activity, and sedentary time were obtained from questionnaires filled by children with support from their parents. Age was calculated as date of examination subtracted by date of birth, and then divided into three age groups: 6~11 years, 12~14 years, and 15~17 years. All areas were divided into urban and rural areas, and further divided into large cities, small to medium cities, general rural areas, and poor rural areas. Household annual income was divided into five categories: <15,000 CNY(Chinese Yuan)/year, 15,000 to <30,000 CNY/year, and ≥30,000 CNY/year. Physical activity information was indicated by variables such as “whether exercises were performed regularly or not,” and “hours spent being sedentary each day”. Children’s fasting height and weight were examined in the early morning, with the children wearing only their underwear, and calculated as BMI (kg/m^2^) to assess overweight and obesity status. Based on the WHO reference population, the Z score of height or BMI was calculated as the actual value minus the median value, divided by the SD for the corresponding age and gender in the WHO reference population. The children were grouped according to their nutritional status: overweight (>1 for BMI Z score) or obesity (>2 for BMI Z score), following the WHO standards and classifications.

### 2.3. Statistical Analyses

All analyses were conducted using SAS 9.3 (SAS Institute Inc., Cary, NC, USA). The Wilcoxon test was used to measure continuous variables (e.g., energy intake), two-categorical variables (e.g., sex). The Kruskal–Wallis test was used to measure multi-categorical variables (e.g., age). Multivariate logistic regression was used to evaluate the association between SSB consumption and obesity status. Model 1 was adjusted for sex, age, area, and household income. Model 2 was adjusted for sex, age, area, household income, physical activity, sedentary time and energy intake, and fat intake and carbohydrate intake. Odds ratios (ORs) and 95% confidence intervals (95% CIs) were calculated. All *p*-values were two-tailed, and *p*-values < 0.05 were considered to indicate statistical significance.

## 3. Results

### 3.1. Demographic Characteristics

In total, 25,553 children were enrolled in this study, which included 10,212 children aged 6~10 years (40.1%), 8851 children aged 11~14 years (34.6%), and 6,90 hildren aged 15~17 years (25.4%). Further, 13,842 (54.2%) and 11,711 (45.8%) children were from urban and rural areas, respectively. The proportions of male and female children were almost equal. Annual household income was low at 15,000 Yuan in 2009 among 49.0% (12,522) of the children. The median energy intake, fat intake and carbohydrate intake in children were 1892.3 kal/day, 63.9 g/day, 269.9 g/day, respectively (as illustrated in Table 1).

### 3.2. SSB Consumption Frequency

Table 2 shows the SSB consumption frequency for all children by geographic area, age group, sex, annual household income level, physical activity, sedentary time, energy intake and SSB type.

In total, the median SSB consumption frequency among Chinese children aged 6~17 years was 2.2 times/week. Approximately 24.5% of the children consumed SSBs <once/week, and 25.9% consumed SSBs ≥5 times/week. Males consumed SSBs 2.3 times/week compared with 2.1 times/week in that of females (*p* < 0.05). Children aged 15~17 years consumed SSBs 4.7 times/week, higher than 4.3 times/week for children aged 11~14 years and 3.5 times/week for children aged 6~10 years (*p* < 0.05). Children from urban areas consumed SSBs 3.0 times/week, compared with 1.8 times/week in that of children from rural areas (*p* < 0.05). Children who did not perform physical activities consumed SSBs 3.0 times/week compared with 2.0 times/week in that of children who performed physical activities regularly (*p* < 0.05). Children who consumed energy ≥2500 kcal/d drank SSBs 4.9 times/week compared with 1.4 times/week in that of children who consumed energy < 1500 kcal/d (*p* < 0.05).

The median consumption frequencies for carbonated, milk, juice, and coffee and tea beverages were 0.5, 0.1, 0.5, and 0.0 times/week, respectively. Among those who consumed carbonated beverages, the consumption frequency was ≥5 times/week in 4.3%, and <once/week in 57.8% of the children. Among children who consumed juice beverages, the consumption frequency was ≥5 times/week in 6.1%, and <once/week in 58.3% of the children.

### 3.3. SSB Consumption Quantity

Table 3 shows the SSB consumption quantity for all children by SSB type, age group, sex, geographic area, annual household income level, physical activity, and sedentary time, energy intake and beverage types.

In total, the quantity of SSBs among Chinese school-aged children was 181.0 g/day. Further, 45.9%, 26.6%, and 27.5% of the children consumed <150 g/day, ≥500 g/day, and 150~<500 g/day, respectively. Males consumed 199.0 g/day versus 168 g/day in females (*p* < 0.05). Children aged 15~17 years consumed 285 g/day, compared with 208 g/day in 11~14-year-old children and 129.5 g/day in children aged 6~10 years (*p* < 0.05). Children from urban areas consumed SSBs of 256 g/day versus 123 g/day in children from rural areas (*p* < 0.05). Children who did not perform physical activities regularly consumed SSBs of 249 g/day, compared with 146 g/day in that of children who regularly engaged in physical activities (*p* < 0.05). Children who consumed energy ≥2500 kcal/day drank SSBs of 500 g/day compared with 89 g/day in that of children who consumed energy < 1500 kcal/day (*p* < 0.05).

The median quantity of carbonated, milk, juice, and coffee and tea beverages consumed each day were 200 g, 50 g, 200 g, and 0 g, respectively. Among those who consumed carbonated beverages, 36.0% and 13.3% of them consumed <150 g/day and ≥500 g/day, respectively. Among those who consumed milk beverages, 37.4% and 20.4% of them consumed <150 g/day and ≥500 g/day, respectively.

### 3.4. SSB Consumption and Overweight/Obesity

In total, 16.5% of the children were overweight and obesity; 21.1% and 11.9% among males and females, respectively (*x*^2^ = 20.731, *p* < 0.05). The prevalence of overweight/obesity in children aged 6~10, 11~14, and 15~17 years were 20.9%, 16.6%, and 9.6%, respectively (*x*^2^ =359.487, *p* < 0.05). In total, 19.3% of the children from urban areas were overweight/obesity, compared with 13.2% in that of children from rural areas (*x*^2^ = −10.341, *p* < 0.05). The prevalence of overweight/obesity in children who did not engage in physical activities was 18.3%, which was higher than that of children who regularly engaged in physical activities (*x*^2^ = 5.181, *p* < 0.05) (as illustrated in Table 4).

Additionally, 18.1% and 13.7% of the children who consumed SSBs ≥5 times/week and <once/week, respectively, were overweight/obesity (*x*^2^ = 26.135, *p* < 0.05). The prevalence of overweight/obesity in children who consumed SSBs ≥500 g/day was 17.2%, which was higher than that of children who consumed <150 g/day (x^2^ = 12.867, *p* < 0.05) (as illustrated in Table 4).

When we included the frequency or quantity of SSB consumption as independent variables in the logic model respectively, after controlling for region, age, sex, annual household income, physical activity, sedentary time, energy intake, fat intake, and carbohydrate intake, children who consumed SSBs more frequently were more likely to be overweight/obesity. The ORs (95%CIs) for “1~<5 times/week” and “≥5 times/week” were 1.133 (1.011~1.268) and 1.147 (1.003~1.312), respectively, compared with that of the reference group (<once/week) (As illustrated in Table 5).

## 4. Discussion

This paper described SSB consumption and its association with obesity among Chinese children aged 6~17 years in 2012, which was highly representative of the national population. In total, Chinese school-aged children consumed 181.0 g/day of SSBs 2.2 times/week. Compared with that of a similar national study in 2002, the proportion of children aged 15~17 years who consumed SSBs more than once per week increased greatly from 14.2% in 2002 to 79.2% in 2012 [19]. The rapid growth of beverage production and supply during the past decades contributed significantly to the growing consumption of SSBs among children. In 2019, the annual beverage production in China reached 177.6 million tons, which was 7% higher than that of the previous year [20]. However, our study found that 25.9% of children consumed SSBs more than 5 times per week in 2012. This proportion declined slightly to 18.9% in 2016, according to the latest report on Chinese Residents’ Chronic Diseases and Nutrition [4]. It revealed that children’s SSB consumption might be affected not only by the development of the beverage industry, but also by marketing and advertisement, parents’ dietary habits, and nutrition education [21,22,23,24,25,26,27]. However, the specific reasons for this should be explored in further studies.

Our study found that older children, males, children from urban areas, and children with higher socioeconomic status were more likely to consume SSBs. These results were consistent with those of other studies in the United States and Europe [24,28,29]. Tasevska et al. analyzed 1403 children from low income cites in United States and found that SSB consumption was higher among children 12~18 years versus 3~5 years [28]. Rosinger A et al. analyzed NHANSE data in youth from 2011~2014 and found that boys consumed 164 kcal from sugar-sweetened beverages versus 121 kcal in girls, and older youth had the highest intake of daily calories from SSBs [29]. We also found that Chinese children preferred juice and milk beverages to carbonated and tea beverages, while children from the United States and other Western countries were more likely to consume carbonated beverages [15,25,30]. These results indicated that teenagers, males, and children from urban areas are key groups that require more attention and interventions on nutrition education and behavior change. At the same time, positive education on topics like “How to choose appropriate drinks” is required for those populations.

Extensive research has shown an association between the increase in SSB consumption and childhood overweight and obesity [31,32,33]. According to the latest report on Chinese Residents’ Chronic Diseases and Nutrition in 2020, the prevalence of overweight/obesity in children and adolescents aged 6~17 years was 19% in 2016, which increased by 5.0% from 2012 [4]. Our study indicated that children who consumed SSBs at a higher frequency and quantity were more likely to be overweight and obesity. For instance, children who consumed SSBs 1~<5 times/week and ≥5 times/week were 11.7% and 12.9%, respectively, more likely to be overweight/obesity than children who consumed SSBs less than once/week. He et al. conducted a study among children aged 7~18 years in South China and indicated that SSB consumers adjusted OR of 2.08 for obesity than non-consumers [33]. Duffey and Poti found that replacing SSB with water could decrease the percent of energy from beverages from 17% to 11% [34]. This indicated a strong relationship between SSB consumption and energy intake. Marshall et al. indicated that additional 8 oz SSB consumed/day increased the BMI z-score an average 0.050 units in childhood and adolescents [8]. Moreover, our study and studies conducted in Spain, Malaysia, and New Caledonia showed that children who did not perform physical activities and had more sedentary time consumed SSBs much more often than their counterparts [27,35,36,37] These results revealed the impact of dietary behavior and physical activity on childhood obesity.

International organizations and experts suggested that reducing SSB consumption may be an effective measure to control the status of overweight and obesity. The World Health Organization recommends detailed measurements to reduce excess SSB intake among children and adolescents, including sugar taxation, restricted advertising and marketing, and effective “front of the pack” warning label system [38]. Studies in Chile and Indonesia showed that a tax of 10–20% on SSBs could lead to a 20% consumption reduction among children and adolescents [39,40,41]. Roberto et al. surveyed 2381 parents in the United States and found that fewer parents chose SSBs for their children in the warning label (40%), compared with the no-label (60%) and calorie label conditions (53%) [42]. This suggests that health-related warning labels on SSBs also helped children and adolescents to reduce their urge to buy SSBs [42,43].

This study has some strengths and limitations. The strengths lie in the fact that the CHNHS is a nationwide nutrition and health database that is highly nationally representative. Limitations include the following: this study was conducted using a cross-sectional database, and the trend of SSB consumption over the years could not be evaluated. Recall bias was inevitable owing to the use of the FFQ.

## 5. Conclusions

SSB consumption increased greatly from 2002 to 2012 for school-aged children in China. Older children, males, and those with higher socioeconomic status were more likely to consume SSBs. Children who consumed higher frequencies of SSBs were more likely to be overweight or obesity. Effective measures including sugar taxation, restrictions on advertising and marketing, and warning labels can reduce excess SSB intake and prevent and control obesity among children and adolescents. We suggest that the Chinese government take action to reduce SSB consumption in school-aged children and create other effective measures to achieve the goal of ending childhood obesity in China.

## Figures and Tables

**Table 1:** Sample characteristics.

Sample Characteristics	N (%)
Total	25,553 (100)
Sex	
Male	12,757 (49.9)
Female	12,796 (50.1)
Age (year)	
6~10	10,212 (40.0)
11~14	8851 (34.6)
15~17	6490 (25.4)
Area	
Urban areas	13,842 (54.2)
Rural areas	11,711 (45.8)
Large cities	6360 (24.9)
Medium to small cities	7482 (29.3)
General rural areas	7646 29.9)
Poor rural areas	4065 (15.9)
Income	
≥30,000 CNY/year ^^^	1903 (7.4)
15,000~<30,000 CNY/year	4737 (18.5)
<15,000 CNY/year	12,522 (49.0)
Physical activity	
yes	14,851 (58.1)
no	10,502 (41.1)
Sedentary time	
<3 h	11,303 (44.2)
≥3 h	14,075 (55.1)
Energy intake (kcal/day) ^#^	1892.3 (1419.7, 2572.6) *
Fat intake (g/day) ^#^	63.9 (44.9, 91.4) *
% energy ^#^	31.0 (25.3, 37.1) *
Carbohydrate intake (g/day) ^#^	269.9 (197.7, 370.1) **
% energy ^#^	57.1 (50.8, 63.2) *

Note: ^^^ CNY is abbreviated form of Chinese Yuan. *^#^* Values are presented as median (Q1, Q3). * *p*-value < 0.05. ** *p*-value < 0.01.

**Table 2:** Frequencies of SSB consumption among Chinese 6–17-year-old children in 2012 (*n* = 25,553).

Sample Characteristics	Frequency per Week, Median (q1, q3)	SSB ^^^ Consumption Frequency, *n* (%)			
		<Once/Week	1-<5 Time/Week	≥5 Time/Week	*Z/*$x^{2}$ (*p*-Value)
**Total**	2.2 (0.1, 5.0)	6250 (24.5)	12,689 (49.7)	6614 (25.9)	
**Sex**					
Male	2.3 (1.0, 5.0)	2996 (23.5)	6239 (48.9)	3522 (27.6)	6.087 (<0.001)
Female	2.1 (1.0, 4.7)	3254 (25.4)	6450 (50.4)	3092 (24.2)	
**Age (year)**					
6~10	3.5 (2.0, 0.9)	2817 (27.6)	5381 (52.7)	2014 (19.7)	357.4 (<0.001)
11~14	4.3 (2.3, 1.0)	2082 (23.5)	4360 (49.3)	2409 (27.2)	
15~17	4.7 (3.0, 1.0)	1351 (20.8)	2948 (45.4)	2191 (33.8)	
**Area**					
Urban areas	3.0 (1.2, 6.0)	2516 (18.2)	6863 (49.6)	4463 (32.2)	30.96 (<0.001)
Rural areas	1.8 (0.7, 4.0)	3734 (31.9)	5826 (49.7)	2151 (18.4)	
Large cities	3.6 (1.6, 7.0)	850 (13.4)	3089 (48.6)	2421 (38.1)	1552.1 (<0.001)
Medium to small cities	2.5 (1.0, 5.0)	1666 (22.3)	3774 (50.4)	2042 (27.3)	
General rural areas	2 (0.9, 4.2)	2041 (26.7)	3934 (51.5)	1671 (21.9)	
Poor rural areas	1.1 (0.5, 2.7)	1693 (41.6)	1892 (46.5)	480 (11.8)	
**Income**					
≥30,000 CNY/year	3.5 (1.5, 6.5)	297 (15.6)	911 (47.9)	695 (36.5)	367.3 (<0.001)
15,000~<30,000 CNY/year	2.7 (1.2, 5.0)	894 (18.9)	2487 (52.5)	1356 (28.6)	
<15,000 CNY/year	2.0(0.9, 4.2)	3582 (28.6)	6190 (49.4)	2750 (22.0)	
**Physical activity**					
yes	2.0 (0.9, 4.0)	4271 (28.8)	7386 (49.7)	3194 (21.5)	23.32 (<0.001)
no	3.0 (1.2, 6.0)	1920 (18.3)	5209 (49.6)	3373 (32.1)	
**sedentary time**					
<3 h	2.0 (0.9, 4.2)	3107 (27.5)	5671 (50.2)	2525 (22.3)	−13.37 (<0.001)
≥3 h	2.5 (1.0, 5.2)	3086 (21.9)	6943 (49.3)	4046 (28.7)	
**Energy intake**					
<1500 kcal/day	1.4 (0.7, 3.0)	2021 (35.1)	3072 (53.4)	664 (11.5)	2198.7 (<0.001)
1500~<2500 kcal/day	2.4 (1.0, 5.0)	1830 (21.6)_	4527 (53.3)	2135 (25.1)	
≥2500 kcal/day	4.9 (2.0, 9.0)	543 (10.3)	2110 (40.1)	2604 (49.5)	
**SSB type**					
Carbohydrate beverages	0.5 (0.1, 1.0)	14,759 (57.8)	9704 (38.0)	1090 (4.3)	
Milk beverages	0.1 (0.0, 1.0)	19,043 (74.5)	5996 (23.5)	514 (2.0)	
Juice beverages	0.5 (0.0, 2.0)	14,902 (58.3)	9100 (35.6)	1551 (6.1)	
Coffee and tea beverages	0.0(0.0, 0.8)	19,371 (75.8)	5118 (20.0)	1064 (4.2)	

Note: ^^^ SSB is short for Sugar-sweetened beverage.

**Table 3:** Quantities of SSB consumption among Chinese 6–17-year-old children in 2012 (*n* = 25,553).

Sample Characteristics	Quantity (g/Day), Median(q1,q3)	SSB Consumption Quantity, *n* (%)			
		<150 g/Day	150~<500 g/Day	≥500 g/Day	Z/$x^{2}$ (*p*-Value)
**Total**	181.0 (54.0, 533.0)	11,726 (45.9)	7030 (27.5)	6797 (26.6)	
**Sex**					
Male	199.0 (59.0, 570.0)	5643 (44.2)	3491 (27.4)	3623 (28.4)	6.5101 (<0.001) *
Female	168.0 (49.0, 493.0)	6083 (47.5)	3539 (27.7)	3174 (24.8)	
**Age (year)**					
6~10	129.5 (41.0, 368.0)	5460 (53.5)	2857 (28.0)	1895 (18.6)	685.2 (<0.001)
11~14	208.0 (60.0, 571.0)	3838 (43.4)	2503 (28.3)	2510 (28.4)	
15~17	285.0 (76.0, 826.0)	2428 (37.4)	1670 (25.7)	2392 (36.9)	
**Area**					
Urban areas	256.0 (78.0, 684.0)	5226 (37.8)	4051 (29.3)	4565 (33.0)	30.51 (<0.001)
Rural areas	123.0 (36.0, 356.0)	6500 (55.5)	2979 (25.4)	2232 (19.1)	
Large cities	324.5 (107.0, 855.0)	2021 (31.8)	1885 (29.6)	2454 (38.6)	1523 (<0.001)
Medium to small cities	210.0 (59.0, 567.0)	3205 (42.8)	2166 (28.9)	2111 (28.2)	
General rural areas	157.0 (53.0, 449.0)	3754 (49.1)	2121 (27.7)	1771 (23.2)	
Poor rural areas	71.0 (25.0, 219.0)	2746 (67.6)	858 (21.1)	461 (11.3)	
**Income**					
≥30,000 CNY/year	279.0 (79.0, 699.0)	702 (36.9)	539 (28.3)	662 (34.8)	201.01 (<0.001)
15,000~<30,000 CNY/year	223.0 (71.0, 570.0)	1938 (40.9)	1444 (30.5)	1355 (28.6)	
<15,000 CNY/year	154.0 (45.0, 456.0)	6185 (49.4)	3423 (27.3)	2914 (23.3)	
**Physical activity**					
yes	146.0 (43.0, 428.0)	7495 (50.5)	4029 (27.1)	3327 (22.4)	20.02 (<0.001)
no	249.0 (71.0, 684.0)	4132 (39.3)	2942 (28.0)	3428 (32.6)	
**sedentary time**					
<3 h	142.0 (43.0, 427.0)	5791 (51.2)	3024 (26.8)	2488 (22.0)	17.09 (<0.001)
≥3 h	220.0 (67.0, 616.0)	5850 (41.6)	3959 (28.1)	4266 (30.3)	
Energy intake					
<1500 kcal/day	89.0 (31.0, 238.0)	3647 (63.4)	1492 (25.9)	618 (10.7)	2402.9 (<0.001)
1500~<2500 kcal/day	199.0 (66.0, 513.0)	3692 (43.5)	2629 (31.0)	2171 (25.6)	
>2500 kcal/day	500.0 (159.0, 1200.0)	1273 (24.2)	1339 (25.5)	2645 (50.3)	
**SSB type**					
Carbohydrate beverages	200.0 (50.0, 275.0)	9208 (36.0)	12,952 (50.7)	3393 (13.3)	
Milk beverages	50.0 (0.0, 200.0)	9550 (37.4)	10,788 (42.2)	5215 (20.4)	
Juice beverages	200.0 (0.0, 400.0)	15,256 (59.7)	8595 (33.6)	1702 (6.7)	
Coffee and tea beverages	0.0 (0.0, 220.0)	16,541 (64.7)	6606 (25.9)	2406 (9.4)	

Note: * *p*-value < 0.05.

**Table 4:** Prevalence of overweight/obesity for different groups among Chinese school aged children in 2012 (*n* = 25,553).

Sample Characteristics	Overweight and Obesity		Z/$x^{2}$	*p*-Value
	No, *n* (%)	Yes, *n* (%)		
Total	21,333 (83.5)	4220 (16.5)		
SSB consumption frequency				
<1 time/week	5392 (86.3)	858 (13.7)	26.135	<0.001
1~<5 time/week	10,525 (82.9)	2164 (17.1)		
≥5 time/week	5416 (81.9)	1198 (18.1)		
SSB consumption quantity				
<150 g/day	9937 (84.7)	1789 (15.3)	12.867	0.002
150~<500 g/day	5765 (82.0)	1265 (18.0)		
≥500 g/day	5631 (82.8)	1166 (17.2)		
Sex				
Male	10,064 (78.9)	2693 (21.1)	20.731	<0.001
Female	11,269 (88.1)	1527 (11.9)		
Age (year)				
6~10	8083 (79.2)	2129 (20.9)	359.487	<0.001
11~14	7380 (83.4)	1471 (16.6)		
15~17	5870 (90.4)	620 (9.6)		
Area				
Urban area	11,169 (80.7)	2673 (19.3)	−10.341	<0.001
Rural area	10,164 (86.8)	1547 (13.2)		
Big city	4999 (78.6)	1361 (21.4)	164.386	<0.001
Middle city	6170 (82.5)	1312 (17.5)		
Regular county	6501 (85.0)	1145 (15.0)		
Poor county	3663 (90.1)	402 (9.9)		
Income				
≥30,000 CNY/year	1472 (77.4)	431 (22.7)	70.108	<0.001
15,000~<30,000 CNY/year	3830 (80.9)	907 (19.2)		
<15,000 CNY/year	10,656 (85.1)	1866 (14.9)		
Physical activity				
yes	12,581 (84.7)	2270 (15.3)	5.181	<0.001
no	8578 (81.7)	1924 (18.3)		
Sedentary time				
<3 h	9450 (83.6)	1853 (16.4)	−0.258	0.796
≥3 h	11,731 (83.3)	2344 (16.7)		
Energy intake				
<1500 kcal/day	4746 (82.44)	1011 (17.56)	1.253	0.534
1500~<2500 kcal/day	7019 (82.65)	1473 (17.35)		
>2500 kcal/day	4306 (81.91)	951 (18.09)		

**Table 5:** Association of SSB consumption and status of overweight and obesity among Chinese 6–17-year-old children in 2012.

Sample Characteristics	Unadjusted		Model1		Model2	
	OR ^^^ (95%CI)	*p*-Value	OR (95%CI)	*p*-Value	OR (95%CI)	*p*-Value
SSB Consumption frequency						
<1 time/week	Ref.		Ref.		Ref.	
1~<5 time/week	1.254 (1.126~1.397)	<0.001	1.136 (1.016~1.270)	0.030	1.133 (1.011~1.268)	0.031
≥5 time/week	1.129 (1.132~1.443)	<0.001	1.142 (1.004~1.299)	0.040	1.147 (1.003~1.312)	0.045
**SSB Consumption quantity**						
<150 g	Ref.		Ref.			
150~<500 g	1.188 (1.076~1.312)	0.001	1.114 (1.005~1.234)	0.040	1.110 (1.000~1.231)	0.049
≥500 g	1.050 (0.946~1.165)	0.359	0.989 (0.886~1.105)	0.848	0.989 (0.881~1.110)	0.853

Note: ^^^ OR is short for Odds Ratio. Model1 was adjusted for sex, age, area, and household income. Model2 was adjusted for sex, age, area, household income, physical activity, sedentary time and energy intake, fat intake, and carbohydrate intake.

## References

[1] WHO (2020). Obesity and Overweight Facts Sheet.

[2] Ogden C.L., Carroll M.D., Lawman H.G., Fryar C.D., Kruszon-Moran D., Kit B.K., Flegal K.M. (2016). Trends in Obesity Prevalence Among Children and Adolescents in the United States, 1988-1994 Through 2013-2014. JAMA.

[3] (2016). China Health and National Planning. Report on Chinese Residents’ Chronic Disease and Nutrition in 2015.

[4] The State Council Information Office Press Conference on 23 December 2020. http://www.nhc.gov.cn/xcs/s3574/202012/bc4379ddf4324e7f86f05d31cc1c4982.shtml.

[5] Twarog J.P., Peraj E., Vaknin O.S., Russo A.T., Baidal J.A.W., Sonneville K.R. (2020). Consumption of sugar-sweetened beverages and obesity in SNAP-eligible children and adolescents. Prim. Care Diabetes.

[6] Hardy L.L., Bell J., Bauman A., Mihrshahi S. (2017). Association between adolescents’ consumption of and different types of sugar-sweetened beverages with oral health impacts and weight status. Aust. N. Z. J. Public Health.

[7] Garduño-Alanís A., Malyutina S., Pajak A., Stepaniak U., Kubinova R., Denisova D., Pikhart H., Peasey A., Bobak M., Stefler D. (2019). Association between soft drink, fruit juice consumption and obesity in Eastern Europe: Cross-sectional and longitudinal analysis of the HAPIEE study. J. Hum. Nutr. Diet..

[8] Marshall T.A., Curtis A.M., Cavanaugh J.E., Warren J.J., Levy S.M. (2019). Child and Adolescent Sugar-Sweetened Beverage Intakes Are Longitudinally Associated with Higher Body Mass Index z Scores in a Birth Cohort Followed 17 Years. J. Acad. Nutr. Diet..

[9] Harrington J.M., Perry C., Keane E., Perry I.J. (2020). Sugar-sweetened beverage consumption and association with weight status in Irish children: A cross-sectional study prior to the introduction of a government tax on sugar-sweetened beverages. Public Health Nutr..

[10] Frantsve-Hawley J., Bader J.D., Welsh J.A., Wright J.T. (2017). A systematic review of the association between consumption of sugar-containing beverages and excess weight gain among children under age 12. J. Public Health Dent..

[11] Miller C., Ettridge K., Wakefield M., Pettigrew S., Coveney J., Roder D., Durkin S., Wittert G., Martin J., Dono J. (2020). Consumption of Sugar-Sweetened Beverages, Juice, Artificially-Sweetened Soda and Bottled Water: An Australian Population Study. Nutrients.

[12] Miller C., Wakefield M., Braunack-Mayer A., Roder D., O’Dea K., Ettridge K., Dono J. (2019). Who drinks sugar sweetened beverages and juice? An Australian population study of behaviour, awareness and attitudes. BMC Obes..

[13] Della Corte K., Fife J., Gardner A., Murphy B.L., Kleis L., Della Corte D., Buyken A.E. (2021). World trends in sugar-sweetened beverage and dietary sugar intakes in children and adolescents: A systematic review. Nutr. Rev..

[14] Wang Y.C., Bleich S.N., Gortmaker S.L. (2008). Increasing caloric contribution from sugar-sweetened beverages and 100% fruit juices among US children and adolescents, 1988–2004. Pediatrics.

[15] Bleich S.N., Vercammen K.A., Koma J.W., Li Z. (2018). Trends in Beverage Consumption among Children and Adults, 2003–2014. Obesity.

[16] Hwang S.B., Park S., Jin G.R., Jung J.H., Park H.J., Lee S.H., Shin S., Lee B.H. (2020). Trends in Beverage Consumption and Related Demographic Factors and Obesity among Korean Children and Adolescents. Nutrients.

[17] Liu A.L., Duan Y.F., Xiao-Qi H.U. (2011). Change in snacking behaviors of children in four cities of China over 10 years. Chin. J. School Health.

[18] National Burea of Statistics (2019). Annual Data: Index for Residents ‘Living. https://data.stats.gov.cn/easyquery.htm?cn=C01.

[19] Zhai F.Y.Y., Yang X.G. (2006). Report on Chinese Nutrition and Health Survey.

[20] SHI DAN LZ (2020). The development status and trend of beverage industry in China. Food Ferment Sci. Technol..

[21] Gui Z.-H., Zhu Y.-N., Cai L., Sun F.-H., Ma Y.-H., Jing J., Chen Y.-J. (2017). Sugar-Sweetened Beverage Consumption and Risks of Obesity and Hypertension in Chinese Children and Adolescents: A National Cross-Sectional Analysis. Nutrients.

[22] Rauzon S., Randel-Schreiber H., Kuo E., Schwartz P., Reed A.L., Thompson H.R. (2020). The association between sugar-sweetened beverage availability in school vending machines and school staff sugar-sweetened beverage consumption. Prev. Med. Rep..

[23] Lundeen E.A., Park S., Onufrak S., Cunningham S., Blanck H.M. (2018). Adolescent Sugar-Sweetened Beverage Intake is Associated with Parent Intake, Not Knowledge of Health Risks. Am. J. Health Promot..

[24] Mazarello Paes V., Hesketh K., O'Malley C., Moore H., Summerbell C., Griffin S., Lakshman R. (2015). Determinants of sugar-sweetened beverage consumption in young children: A systematic review. Obes. Rev..

[25] Pawellek I., Grote V., Theurich M., Closa-Monasterolo R., Stolarczyk A., Verduci E., Xhonneux A., Koletzko B., European Childhood Obesity Trial Study Group (2016). Factors associated with sugar intake and sugar sources in European children from 1 to 8 years of age. Eur. J. Clin. Nutr..

[26] Bolt-Evensen K., Vik F.N., Stea T.H., Klepp K.-I., Bere E. (2018). Consumption of sugar-sweetened beverages and artificially sweetened beverages from childhood to adulthood in relation to socioeconomic status—15 years follow-up in Norway. Int. J. Behav. Nutr. Phys. Act..

[27] Gan W.Y., Mohamed S.F., Law L.S. (2019). Unhealthy Lifestyle Associated with Higher Intake of Sugar-Sweetened Beverages among Malaysian School-Aged Adolescents. Int. J. Environ. Res. Public Health.

[28] Tasevska N., DeLia D., Lorts C., Yedidia M., Ohri-Vachaspati P. (2017). Determinants of Sugar-Sweetened Beverage Consumption among Low-Income Children: Are There Differences by Race/Ethnicity, Age, and Sex?. J. Acad. Nutr. Diet..

[29] Rosinger A., Herrick K., Gahche J., Park S. (2017). Sugar-sweetened Beverage Consumption among U.S. Youth, 2011–2014. NCHS Data Brief..

[30] Pereira R.A., Souza A.M., Duffey K.J., Sichieri R., Popkin B.M. (2015). Beverage consumption in Brazil: Results from the first National Dietary Survey. Public Health Nutr..

[31] Viner R.M., Kinra S., Nicholls D., Cole T., Kessel A., Christie D., White B., Croker H., Wong I.C.K., Saxena S. (2017). Burden of child and adolescent obesity on health services in England. Arch. Dis. Child..

[32] Shin S., Kim S.-A., Ha J., Lim K. (2018). Sugar-Sweetened Beverage Consumption in Relation to Obesity and Metabolic Syndrome among Korean Adults: A Cross-Sectional Study from the 2012–2016 Korean National Health and Nutrition Examination Survey (KNHANES). Nutrients.

[33] He B., Long W., Li X., Yang W., Chen Y., Zhu Y. (2018). Sugar-Sweetened Beverages Consumption Positively Associated with the Risks of Obesity and Hypertriglyceridemia Among Children Aged 7–18 Years in South China. J. Atheroscler. Thromb..

[34] Duffey K.J., Poti J. (2016). Modeling the Effect of Replacing Sugar-Sweetened Beverage Consumption with Water on Energy Intake, HBI Score, and Obesity Prevalence. Nutrients.

[35] Bibiloni MD M., Özen A.E., Pons A., González-Gross M., Tur J.A. (2016). Physical Activity and Beverage Consumption among Adolescents. Nutrients.

[36] Wattelez G., Frayon S., Cavaloc Y., Cherrier S., Lerrant Y., Galy O. (2019). Sugar-Sweetened Beverage Consumption and Associated Factors in School-Going Adolescents of New Caledonia. Nutrients.

[37] WHO (2016). Report of the Commission on Ending Childhood Obesity.

[38] WHO (2015). Fiscal Policies for Diet and Prevention of no Communicable Diseases: Technical Meeting Report.

[39] Taillie L.S., Reyes M., Colchero M.A. (2020). An evaluation of Chile's Law of Food Labeling and Advertising on sugar-sweetened beverage purchases from 2015 to 2017: A before-and-after study. PLoS Med..

[40] GSM (2018). Report on the Consumption of Sugar-Sweetened Beverages of Children in China.

[41] Roberto C.A., Wong D., Musicus A., Hammond D. (2016). The Influence of Sugar-Sweetened Beverage Health Warning Labels on Parents’ Choices. Pediatrics.

[42] Schillinger D., Jacobson M.F. (2016). Science and Public Health on Trial: Warning Notices on Advertisements for Sugary Drinks. JAMA.

[43] VanEpps E.M., Roberto C.A. (2016). The Influence of Sugar-Sweetened Beverage Warnings: A Randomized Trial of Adolescents' Choices and Beliefs. Am. J. Prev. Med..

